# Editorial for the Special Issue on New Trends and Applications in Femtosecond Laser Micromachining

**DOI:** 10.3390/mi13020150

**Published:** 2022-01-19

**Authors:** Francesca Bragheri, Petra Paiè, Rebeca Martínez Vázquez

**Affiliations:** Istituto di Fotonica e Nanotecnologie (IFN)—CNR, Piazza L. da Vinci 32, 20133 Milano, Italy; francesca.bragheri@ifn.cnr.it (F.B.); petra.paie@polimi.it (P.P.)

Femtosecond laser micromachining is becoming an established fabrication technique for transparent material processing in three dimensions [1]. Laser writing permits obtaining different material modifications, depending on the laser parameters as well as on the material properties. For instance, it allows creating micrometer-size structures by two-photon polymerization (additive approach), as well as creating empty channels inside the material (subtractive approach). On the other hand, transformative approaches are also permitted, thus locally modifying the material properties for optical waveguide fabrication and material welding. Therefore, the capabilities of femtosecond laser micromachining open the doors to a plethora of applications ranging from the biological to the information technology field.

This Special Issue is composed of 10 contributions, including original research and reviews. The review paper from Butkute et al. [2] reports a brief but exhaustive overview of the significant advancements in the femtosecond laser machining of glasses, reviewing the possible modifications of the material and highlighting application examples such as optical waveguiding and microfluidic systems. Following the unique capabilities of femtosecond laser technologies, we highlight four macro areas of applications in which we classified the contributions of the issue.

**Material processing**. In this category, we include papers that discuss the use of femtosecond lasers either to structure or to process the substrates of interest. Most of the previously mentioned approaches can be exploited for the precise material structuring. Umenne [3] exploits the femtosecond laser ablation of YBCO thin films to fabricate sub-micron and nano-sized bridges to be used as Josephson junctions. A new irradiation method based on a double pulse approach has been proposed by Stankevic et al. [4] to optimize the realization of hollow structures in bulk fused silica. The irradiation with two orthogonally polarized laser beams leads to the formation of nanogratings with grid-like morphology, giving rise to an improvement of chemical etching anisotropy and to a faster processing speed. Microfractures produced by femtosecond (FS) lasers have been exploited by Gaudiuso et al. [5] to achieve stealth dicing of quartz with neat and flat cut edges in a single pass. The result is ascribed to tensile stresses that after relaxation produce microfractures that are guided by the laser to achieve cutting.

**Lab-on-chip**. Here, we include papers that demonstrate the potential of the use of femtosecond lasers in the bio-chemical fields. Microfluidic platforms are widely used and can be realized by diverse technologies either in plastic or in glass. Anyway, femtosecond laser micromachining still plays a key role for its intrinsic three-dimensional capabilities as demonstrated by Qi et al. [6] who realized an efficient 3D mixer based on Baker’s transformation principle that could find applications in microfluidic synthesis of materials or fine chemistry microreactions. The device is realized by femtosecond irradiation followed by chemical etching and hydroxide-catalysis bonding in fused silica. Different applications are envisaged for the reservoir etched in borosilicate glass by high-fluence FS lasers by Owusu-Ansah [7] et al. In their work, they optimize the etching depth as a function of fluence and fabricate multi-depth reservoirs with large areas for possible application in microfluidic transport investigation or CO_2_ storage.

**Biophotonics**. In this category, we decided to include papers where femtosecond lasers are used for machining or analysis specifically in the biomedical field. Keleman et al. [8] exploited two-photon polymerization for the fabrication of microtools that thanks to their three-dimensional shape could be easily actuated by optical tweezer and exploited to probe single cell elasticity, without inducing photodamage to the sample. FS-laser technology in the medical field is used also as precise and reliable tool, for example, in ophthalmic surgery. As reported in the review by Latz et al. [9] some of the applications in this field still require research and development, while operations such as FS-laser-assisted cataract and corneal surgery have reached highly standardized levels worldwide. 

**Integrated optics**. In this category, we include papers where laser modifications are exploited to fabricate optical waveguides or other integrated optical components. A very active application field is the one concerning quantum optics; in this contest, the paper by Li et al. [10] reports a photonic quantum chip fabricated in borosilicate glass by femtosecond laser transformative approach. The high quality of the chip encompassing a Hadamard and a CNOT gate to generate a four path-encoded Bell states is demonstrated by the high value of the fidelity of the reconstructed truth table. Femtosecond laser irradiation followed by chemical etching is instead used by Sala et al. [11] for the fabrication of micromirrors that could be included in integrated photonic or optofluidic chips. The effects of thermal annealing in reducing the residual roughness are evaluated with a profilometer to optimize the smoothening performed by the oven.

We would like to thank all the contributors for submitting their papers to this Special Issue. We also thank all the reviewers for dedicating their time to help improve the quality of the submitted papers.
